# Dietary n-3 Fatty Acid, α-Tocopherol, Zinc, vitamin D, vitamin C, and β-carotene are Associated with Age-Related Macular Degeneration in Japan

**DOI:** 10.1038/srep20723

**Published:** 2016-02-05

**Authors:** Aya Aoki, Maiko Inoue, Elizabeth Nguyen, Ryo Obata, Kazuaki Kadonosono, Shoji Shinkai, Hideki Hashimoto, Satoshi Sasaki, Yasuo Yanagi

**Affiliations:** 1 Department of Ophthalmology, Graduate School of Medicine and Faculty of Medicine, The University of Tokyo, Tokyo, Japan; 2 Yokohama City University Medical Center, Yokohama, Kanagawa, Japan; 3 Department of Social and Preventive Epidemiology, School of Public Health, The University of Tokyo, Tokyo, Japan; 4 Tokyo Metropolitan Institute of Gerontology, Tokyo, Japan; 5 Department of Health Economics and Epidemiology Research, University of Tokyo School of Public Health, Tokyo, Japan; 6 Singapore National Eye Center, 11 Third Hospital Ave, Singapore 168751; 7 Singapore Eye Research Institute, 11 Third Hospital Ave, Singapore 168751

## Abstract

This case-control study reports the association between nutrient intake and neovascular age-related macular degeneration (AMD) in Japan. The nutrient intake of 161 neovascular AMD cases from two university hospitals and 369 population-based control subjects from a cohort study was assessed using a brief-type self-administered questionnaire on diet history, which required respondent recall of the usual intake of 58 foods during the preceding month. Energy-adjusted nutrient intake values were compared between the groups. Logistic regression analysis was used to estimate odds ratios (ORs) and 95% CIs adjusted for smoking history, age, sex, chronic disease history, supplement use, and alcohol consumption. Logistic regression analysis demonstrated that low intakes of n-3 fatty acid, α-tocopherol, zinc, vitamin D, vitamin C, and β-carotene were associated with neovascular AMD (Trend P < 0.0001 for n-3 fatty acid, Trend P < 0.0001 for α-tocopherol, Trend P < 0.0001 for zinc, Trend P = 0.002 for vitamin D, Trend P = 0.04 for vitamin C, Trend P = 0.0004 for β-carotene). There was no association with retinol or cryptoxanthin intake and neovascular AMD (P = 0.67, 0.06).

Age-related macular degeneration (AMD) has a strong genetic component; however, modifiable factors such as smoking history, antioxidant supplement intake, and diet are also associated with slower progression of early- to advanced-stage neovascular AMD^1^,^2^,^3^. Among nutritional factors, there is consistent evidence from a decade of epidemiologic observations in several populations that high intake of n-3 polyunsaturated fatty acid, present in oily fish, is associated with reduced risk^4^,^5^,^6^. A previous case-control study conducted as part of the Eye Disease Case Control Study (United States) demonstrated an association between higher intake of n-3 fatty acids and lower risk of advanced AMD among individuals^4^, while the Blue Mountains Eye Study (Australia) demonstrated a protective effect of n-3 fatty acids in late AMD^5^. Moreover, the participants in the Age-Related Eye Disease Study (AREDS) who consumed the highest level of n-3 fatty acids were shown to be significantly less likely to have neovascular AMD at baseline^6^. A population-based cross-sectional study further identified an inverse association between dietary consumption of docosahexaenoic acid (DHA) and of eicosapentaenoic acid (EPA) and neovascular AMD. It has therefore been postulated that high intake of dietary n-3 fatty acids can reduce the risk or slow the progression of AMD development^7^,^8^. In line with this assumption, consumption of fatty fish such as salmon and tuna has been shown to increase serum n-3 fatty acid concentrations^9^.

Other nutritional factors thought to be related to neovascular AMD are consumption of β-carotene, vitamin C, vitamin E (α-tocophemol), and zinc^10^, although this association remains controversial, with some studies showing a significant relationship but others not^11^. In addition to these nutrients, several case-control studies have demonstrated an association between vitamin D and neovascular AMD^11^,^12^.

Recently, the prevalence of neovascular AMD is thought to have drastically changed^13^. For example, the Specified Disease Conference, an advisory body to the Health Service Bureau of the Japanese Ministry of Health, Labor and Welfare that regularly investigates causative diseases indicated on physical disability certificates issued in Japan, reported that AMD, which did not register in 1994, is now the fourth-leading cause of visual disability, following diabetic retinopathy, glaucoma, and retinitis pigmentosa. However, although the prevalence of neovascular AMD in Japan has increased, it remains relatively low compared with Western countries^14^,^15^. Populations that habitually consume shellfish and lean fish reportedly have elevated n-3 fatty acid levels^16^. It is therefore possible that the uniquely high levels of fish consumption in Japan may contribute to the lower incidence of neovascular AMD. Thus far, however, to the best of our knowledge, no study has examined the relationship between dietary nutrient intake and neovascular AMD in any non-Western population. The object of this study is thus to investigate the association between nutrient intake and neovascular AMD in Japan while capturing the sizable diversity in dietary nutrient intake within the Japanese population.

## Results

Table 1 shows the clinical characteristics of the study participants and their medical history. The mean ± standard deviation for age was 74 ± 7 years for case subjects and 73 ± 6 years for controls (Table 1). The case subjects showed less hyperlipidemia than control subjects (p = 0.04). Smoking history, alcohol consumption, and chronic disease history were not significantly different between the two groups.

As shown in Table 2, the average energy intake and energy-adjusted intake of n-3 fatty acid, α-tocopherol, zinc, vitamin D, vitamin C, and β-carotene was significantly lower in the neovascular AMD group compared to the controls. The results of logistic analysis adjusted for smoking history, age, sex, chronic disease history, supplement use, and alcohol consumption between neovascular AMD and dietary macronutrient intake are shown in Table 3. Low intake of n-3 fatty acid, α-tocopherol, zinc, vitamin D, vitamin C, and β-carotene was found to be associated with neovascular AMD (odds ratios [ORs] for the second, third, fourth and highest quintiles: 1.1 [95% CI, 0.6–1.8], 0.4 [0.2–0.7], 0.6 [0.4–1.1], 0.2 [0.1–0.4] (Trend P < 0.0001) for n-3 fatty acid; 0.6 [0.3–1.0], 0.2 [0.1–0.4], 0.3 [0.2–0.6], 0.2 [0.1–0.3] (Trend P < 0.0001) for α-tocopherol; 0.6 [0.4–1.1], 0.4 [0.2–0.8], 0.2 [0.1–0.4], 0.1 [0.1–0.2] (Trend P < 0.0001) for zinc; 0.6 [0.3–1.1], 0.5 [0.3–0.9], 0.4 [0.2–0.7], 0.4 [0.2–0.8] (Trend P = 0.002) for vitamin D; 0.5 [0.3–0.9], 0.4 [0.2–0.8], 0.5 [0.3–1.0], 0.4 [0.2–0.8] (Trend P = 0.002) for vitamin C; 0.5 [0.3–0.9], 0.4 [0.2–0.8], 0.6 [0.3–1.0], 0.2 [0.1–0.5] (Trend P = 0.002) for β-carotene. There was a downward trend in neovascular AMD for dietary n-3 fatty acid, α-tocopherol, zinc, vitamin D, and β-carotene intake, with a threshold in the significant protective effect for vitamin C; there was no apparent association with retinol or cryptoxanthin intake.

## Discussion

There was a statistically significant difference in dietary intake of n-3 fatty acid, α-tocopherol, zinc, vitamin D, vitamin C, and β-carotene between case subjects and controls. In line with this, multivariable logistic regression showed a lower risk of AMD with higher intakes of n-3 fatty acid, α-tocopherol, zinc, vitamin D, vitamin C, and β-carotene. These findings suggest that higher dietary intake of n-3 fatty acid, α-tocopherol, zinc, vitamin D, vitamin C, and β-carotene could contribute to a reduced risk of neovascular AMD in elderly patients in Japan.

Recent studies have identified an inverse association between the dietary consumption of n-3 fatty acid and neovascular AMD^6^,^7^,^8^. However, no study has investigated the effect of n-3 fatty acid intake at higher doses than those included in the typical Japanese diet on neovascular AMD. A previous case-control study conducted as part of the Eye Disease Case Control Study (United States) demonstrated an association between higher intake of n-3 fatty acids and lower risk of advanced AMD among individuals on a diet low in linoleic acid^4^, while the Blue Mountains Eye Study (Australia) demonstrated a protective effect of n-3 fatty acids in late AMD among those in the highest quintile of intake (OR, 0.18; 95% CI, 0.02–1.38)^5^. Participants in AREDS reporting the highest consumption of n-3 fatty acid were also significantly less likely to have neovascular AMD at baseline^6^. It is worth noting that the median dietary intake of n-3 fatty acid in the lowest and highest quintiles in the United States study (Eye Disease Case Control Study) was 1.02 g/d and 1.16 g/d, respectively^4^, while in the Australian study values ranged from 0.05–0.26 g/day (lowest quartile) to 0.52–2.11 g/day (highest quartile)^5^. In the current study, subjects consumed an average of 3.2 g n-3 fatty acid/day, which is considerably higher than in these previous studies. This is likely due to the higher intake of fish in the Japanese diet compared to diets in other developed countries. Even at this high range of dietary n-3 fatty acid intake, the results showed an inverse relationship between high intake and AMD, suggesting a lower incidence of neovascular AMD with increased n-3 fatty acid intake. However, in AREDS2, the addition of DHA + EPA to the original AREDS supplement formulation (vitamin C, vitamin E, β-carotene, zinc, and copper) did not further reduce the risk of AMD^17^. As discussed, this might have been due to an inadequate dose, inadequate duration of treatment, or both^17^.

Vitamin C and E are non-enzymic antioxidants that protect against oxidative stress, an important contributory factor in the progression of neovascular AMD, with some small case-control studies showing an inverse relationship between serum α-tocopherol levels and neovascular AMD^18^,^19^; the current study demonstrates that dietary intake of α-tocopherol and of vitamin C was associated with a reduced risk of neovascular AMD. However, there seems to be a limit threshold, and, therefore, further studies with larger samples are needed to address whether vitamin C alone is sufficient to reduce the risk of neovascular AMD. AREDS demonstrated that intake of high doses of vitamins C and E and β-carotene alone was not sufficient, but rather required the addition of zinc to reduce the risk of neovascular AMD^10^, the 400-IU dose of vitamin E recommended by AREDS being about 13 times the recommended dietary allowance (RDA). It is worth mentioning that such levels of vitamin E can only be obtained by supplementation.

Zinc is known to be a co-factor of many metabolically active enzymes within the eye, including superoxide dismutase and catalase^20^, which are important in protecting the retina from oxidative damage. Zinc also binds complement factor H, inducing large multimeric forms that lose complement component 3b inhibitory activity^21^, theoretically reducing the risk of neovascular AMD by suppressing chronic inflammation at the retinal pigment epithelium/choroidal interface. Elderly patients are at higher risk of zinc deficiency^22^, which may increase their risk of vision loss from AMD. To date, reports on zinc have varied, showing no significant relationship with neovascular AMD to its protective role^23^. In the current study, there was a downward trend between dietary zinc intake and neovascular AMD, supporting the assertion of the presence of protective effects of zinc in neovascular AMD. It should be noted that a high dose of zinc (80 mg) alone, which again can be obtained only by supplementation, was found to be sufficient to reduce the risk of progression to neovascular AMD in the AREDS cohort.

Vitamin D was found to reduce the risk of early AMD and to have anti-inflammatory properties in several studies^24^. A number of genes involved in inflammatory immune responses are associated with AMD. The current study demonstrated that vitamin D is also associated with neovascular AMD. Fish is a source of vitamin D as well as n-3 fatty acids^25^, and thus, increased fish intake may be a confounding factor.

There was a significant association between β-carotene and neovascular AMD, with the dietary intake of β-carotene in the current population. β-Carotene is contained in plant products and is the most important carotenoid in the human diet. It functions as an antioxidant, reducing oxidative damage and thereby theoretically reducing the risk of neovascular AMD. However, the association between β-carotene and neovascular AMD remains under debate. In the Eye Disease Case-Control Study, investigators found that high dietary intake of carotenoids was associated with a lower risk of advanced AMD. However, the Blue Mountains Eye study found no association between serum β-carotene and AMD. Furthermore, a study of smokers in Finland found that supplementation with β-carotene did not appear to be protective against AMD. Another study suggested a joint effect of dietary β-carotene intake, smoking history, and serum total cholesterol–to–HDL-C ratio, along with nut consumption^26^. AREDS2 reported that elimination of high-dose β-carotene from the original formulation did not affect the incidence of neovascular AMD^17^.

Despite its findings, this study has several limitations that should be considered. First, due to the cross-sectional–observational nature of the study design, it is possible that the case subjects may have changed their dietary habits after diagnosis, leading to a reverse-causality problem. However, those cases that reported a recent change in dietary habits were excluded from the analysis to minimize such bias. Second, the generalizability of our results requires confirmation in future studies, with recruitment of case subjects from a wider range of macular outpatient clinics across Japan. Third, due to the low participation rate and non-ignorable attrition in the Hatoyama Cohort, the included controls may not have been fully representative of the elderly Japanese population, leading to selection bias. Fourth, the age ranges of the case and control subjects were different, though the difference was not significant. Fifth, the diet history questionnaire only allowed respondents to rank their consumption of different foods, not to report absolute nutrient intakes, causing potential for underreporting. The results therefore require clarification in a larger prospective cohort study to avoid any bias in this regard as well.

In conclusion, using a brief-type diet history questionnaire (BDHQ) in a case-control study of AMD, we demonstrated that a high dietary intake of n-3 fatty acid, α-tocopherol, zinc, vitamin D, vitamin C, and β-carotene is associated with a reduced risk of AMD. This study will contribute to further research on the relationship between nutritional supplements and AMD in Japan. Future plans include a case-control study on dietary intake between subjects with different types of AMD (typical AMD, polypoidal choroidal vasculopathy, and retinal angiomatous proliferation) and healthy subjects, and the expansion of the project to other macular outpatient clinics across Japan.

## Materials and Methods

### Ethics statement

This study followed a cross-sectional design. The study protocol was reviewed and approved by the institutional review boards of the University of Tokyo Hospital and Yokohama City University Hospital, and informed consent was obtained from all patients prior to participation. The study protocol of the Hatoyama Cohort Study was reviewed and approved by the Ethical Committee of Tokyo Metropolitan Institute of Gerontology, and written informed consent was also obtained from all participants. All the methods were carried out in accordance with the approved guidelines.

### Subjects

#### Case subjects

Case subjects comprised patients who regularly (here defined as monthly to once every six months, depending on disease activity) visited the macular outpatient clinics of the University of Tokyo Hospital or Yokohama City University Hospital and who were undergoing treatment for AMD. Specific inclusion criteria were as follows: regular visits between June and October 2011 to the University of Tokyo Hospital or between July to September 2012 to Yokohama City University Hospital; diagnosis of neovascular AMD; and age of 50 years or older. Patients were excluded if they had only early or geographic atrophy.

All patients were referred to one of the two hospitals, meaning that all patients were from the local area (Tokyo, Saitama, and Kanagawa Prefectures) and all visited their macular outpatient clinic regularly. During regular visits, patients were made aware of the project and its purpose and were asked if they would be willing to participate. Informed consent was obtained from 206 patients at the University of Tokyo Hospital and 110 patients at Yokohama City University Hospital.

#### Control subjects

All subjects in the control group consisted of individuals aged 65 or over, dwelling in the community in the suburban town of Hatoyama, Saitama Prefecture, as part of the Hatoyama Cohort Study. Hatoyama is located 50 km northwest of central Tokyo and was developed as a commuter town. The aim of the study was to analyze (this aspect of) the health of the aging elderly population within Japan and to facilitate investigation of policies that could be administered to improve elderly health. Details of the Hatoyama Cohort study is described in a previous report^27^.

The control population was recruited first by stratified sampling of 4 groups (age: 65–74 and 75–84 years; residential area: old town and new town). Those with long-term care certification (levels 1–5) and/or living in a nursing home or admitted to hospital were excluded. Since one group (65–74 years, new town) was larger than the other three groups, random sampling was used for this group; for the remaining three groups, a complete census was employed. The 2697 randomly selected participants were mailed a recruitment brochure, explaining the study, its purpose, methods, survey items, and the merits of participating. Recruitment was also performed via the Hatoyama town bulletin to broaden the subject pool.

In total, 742 (27.5%) healthy Japanese individuals over 65 years old volunteered to participate in the baseline study. Among these original participants, 596 (80.3%) participated in the 2-year follow-up examinations in September 2012 As part of the two-year follow-up survey of the Hatoyama Cohort Study, a brief diet history questionnaire was administered (with the goal of determining the dietary habits of the elderly in Japan).

#### Final subjects

In both groups, subjects with no formal ID or an incomplete disease history were excluded (49 in the case group and 5 controls). Those with an extremely high or low energy intake were also excluded (11 in the case group and 18 controls). An extremely low energy intake was defined as less than half the energy requirement for the lowest physical activity category (for men: aged 50–69 years < 1050 (2100 × 0.5) kcal/day, aged 70 and over < 925 (1850 × 0.5) kcal/day; for women: aged 50–69 < 825 (1650 × 0.5) kcal/day, aged 70 and over < 725 (1450 × 0.5) kcal/day), while extremely high energy intake was defined as 1.5 times the energy requirement of the highest physical activity category (for men: aged 50–69 years > 4200 (2800 × 1.5) kcal/day, aged 70 and over > 3750 (2500 × 1.5) kcal/day; for women: age 50–69 > 3300 (2200 × 1.5) kcal/day, age 70 and over > 3000 (2000 × 1.5) kcal/day) according to the Recommended Dietary Allowance for Japanese.

**Figure 1:**
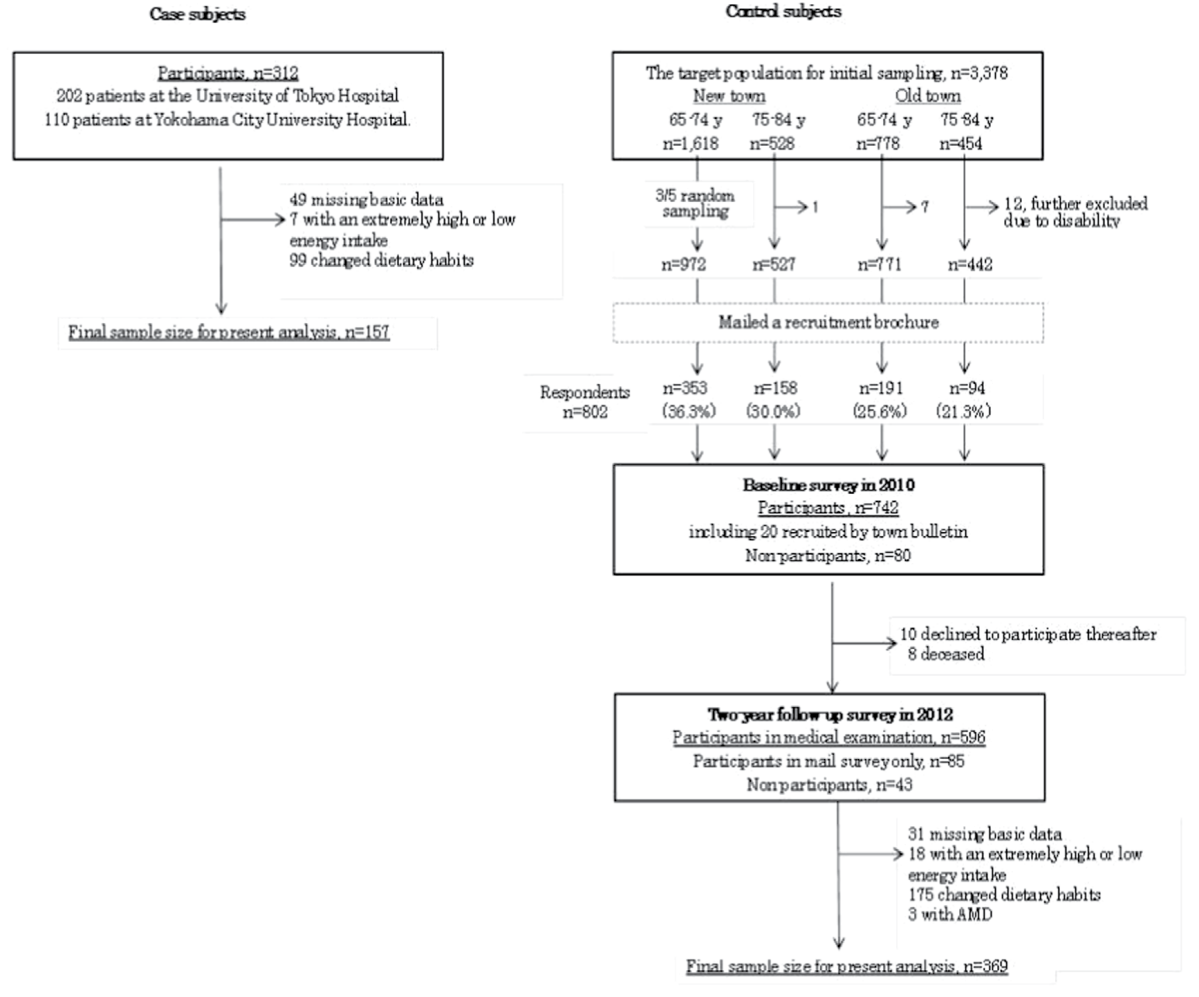
Flowchart describing both case and control subjects who were included and excluded from the analysis.

Subjects who changed their dietary habits (94 case subjects, 157 controls), and those following dietary instructions from a nutritionist or doctor (5 case subjects, 18 controls) were not used in the final analysis. Three control subjects were excluded due to a diagnosis of AMD, and 26 because they did not have matching BDHQ (3) or health check information (23). As a result, a final total of 157 case subjects and 369 controls were included in the analysis (see Fig. 1).

### Dietary assessment

#### Diet history questionnaire

A brief-type, self-administered diet history questionnaire (BDHQ) was used to assess food intake during the previous month. The BDHQ was modified based on the self-reported diet history questionnaire (DHQ), which was evaluated and compared with a 3-day diet record and serum biomarkers^28^. The BDHQ includes 58 food and beverage items. The subjects indicated their mean frequency of consumption in terms of the specified serving size by checking one of seven frequency categories, ranging from “almost never” to “two or more times a day.” The dietary intake estimates for total energy and various nutrients, including n-3 fatty acid, α-tocopherol, zinc, and vitamin D, were calculated using an ad hoc algorithm developed for the BDHQ based on the Standard Tables of Food Composition in Japan. Validation of ranking of energy-adjusted nutrient intake was previously conducted in an adult Japanese population^28^,^29^,^30^,^31^,^32^,^33^.

The BDHQ was distributed to case subjects upon recruitment and returned by post within approximately one month. Any questions regarding how to fill out the questionnaire were taken by phone by the doctors in charge at each hospital. Once collected, the questionnaires were checked for any possible errors and the patients were called to confirm the content.

Control subjects from the Hatoyama Cohort Study were asked to complete the BDHQ during the follow-up study. Because only 599 subjects were included in the follow-up, and due to a lack of information on the subjects lost, a comparison was performed of characteristics at baseline and at follow-up. Three subjects did not return the BDHQ, leaving a final total of 596 participants. The male-to-female ratio was almost the same between baseline and follow-up. The follow-up subjects were slightly older than at baseline, and there was a higher number of participants with a normal body mass index in the follow-up (18.5–25.0).

#### Health status

During their regular hospital visits, case subjects were asked to fill out a short questionnaire on their health status. Questions were asked related to the subject’s status with regard to hypertension, diabetes, hyperlipidemia, cardiovascular disease, stroke, smoking history (current smoker, past smoker, non-smoker; and if a smoker, the number of cigarettes consumed a day and for how many years), and age.

All control subjects were subjected to a health check-up at Hatoyama Health Center during the month of September 2012, as part of the follow-up study. During this check-up, (any) history of physician-diagnosed disease, medication, smoking, alcohol consumption, diet, sleeping, and physical activity was obtained. All items were measured by a physician, public health nurse, or and registered nurse. History of physician-diagnosed disease and smoking were used in the case-control study.

### Statistical analysis

All statistical analyses were conducted using the SAS statistical software package, version 9.3 (SAS Institute Inc., Cary, NC, USA). The residual model was used for energy adjustment^34^. The percentage contribution of dietary nutrients was calculated by dividing the daily total energy intake for each group (case and control subjects) before being combined using the student’s t-test and logistic regression analysis.

With the student’s t-test, significance was determined using the two-sided t-test, with significance set at p = 0.05. The student’s t-test was performed to compare dietary intake values taken from the BDHQ between the case group and controls. For logistic regression, quintiles were used to look at the odds ratios (OR) for risk of AMD, with confidence intervals (CI), between different intake levels. The first quintile was set as the reference level (the lowest). Trends of association were examined using a logistic regression model with scores assigned to the intake level. The following variables, which are known to be confounding factors for AMD, were adjusted for in this study: age (years, continuous), sex (female/male), smoking history (past or current smoker/nonsmoker), supplement use (yes/no), alcohol consumption (yes/no) and chronic disease history (any of the following: hypertension, diabetes, hyperlipidemia, cardiovascular disease, stroke; yes/no). A dummy variable was used for all categorical variables (0 or 1; for sex, 1 or 2).

## Additional Information

**How to cite this article**: Aoki, A. *et al.* Dietary n-3 Fatty Acid, α-Tocopherol, Zinc, vitamin D, vitamin C, and β-carotene are Associated with Age-Related Macular Degeneration in Japan. *Sci. Rep.* **6**, 20723; doi: 10.1038/srep20723 (2016).

## Figures and Tables

**Table 1:** Basic characteristics of the case subjects and controls.

Variable	AMD (n = 157)	Control (n = 369)	p-value
Male (n [%])	106 (67.5)	227 (61.5)	0.2
Age (years)	73.5 ± 7.1	73.1 ± 5.6	0.4
Height (cm)	160.3 ± 8.1	158.1 ± 8.3	1.0
Weight (kg)	58.7 ± 10.4	57.9 ± 9.4	0.8
Body mass index (kg/m^2^)	22.7 ± 2.9	23.1 ± 2.8	0.09
Smoking history (n [%])	91 (58.0)	184 (49.9)	0.09
Hypertension (n [%])	81 (51.6)	181 (49.1)	0.6
Diabetes (n [%])	17 (10.8)	32 (8.7)	0.4
Hyperlipidemia (n [%])	30 (19.1)	101 (27.4)	0.04
Cardiovascular disease (n [%])	7 (4.5)	9 (2.4)	0.3
Cerebral infarction (n [%])	4 (2.6)	20 (5.4)	0.1

Note: All values are n (%) or mean ± SD. AMD, Age-related macular degeneration.

**Table 2:** Energy-adjusted values for specific nutrient intake per day among the case subjects and controls.

Variable (per day)	AMD (n = 157)	Control (n = 369)	p-value
Zinc (mg/d)	8.4 ± 1.3	9.4 ± 1.4	<0.0001
Retinol (μg/d)	479.2 ± 407.1	526.9 ± 394.8	0.2099
Vitamin D (μg/d)	18.3 ± 10.9	21.2 ± 11.5	0.0083
α-tocopherol (mg/d)	7.8 ± 2.2	9.2 ± 2.2	<0.0001
Vitamin C (mg/d)	132.0 ± 60.6	147.2 ± 58.8	0.0074
N-3 fatty acid (g/d)	2.9 ± 0.8	3.3 ± 0.9	<0.0001
β-carotene (μg/d)	3547.0 ± 2105.9	4657.4 ± 2688.9	<0.0001
Cryptoxanthin (μg/d)	379.1 ± 376.5	341.9 ± 297.3	0.2
Average energy intake (kcal/d)	1916.6 ± 564.1	2052.9 ± 559.4	0.011

Notes: Energy-adjusted values were calculated using the residual model.

Student’s t-test, significant difference noted at p = 0.05.

Notes: All values are mean ± SD.

**Table 3:** Associations between dietary intake levels of specific nutrients and neovascular AMD*.

Variable (per day)	Odds ratio (CI)					Trend P
	Q1 (Lowest) (n = 105)	Q2 (n = 105)	Q3 (n = 106)	Q4 (n = 105)	Q5 (Highest) (n = 105)	
Zinc (mg/d)	≤8.0	8.0–8.8	8.8–9.4	9.4–10.2	≥10.2	<0.0001
n of cases/controls	53/52	43/62	33/73	17/88	11/94	
Multivariate adjusted model**	1 (Reference)	0.6 (0.4, 1.1)	0.4 (0.2, 0.8)	0.2 (0.1, 0.4)	0.1 (0.1, 0.4)	
Retinol (μgram/d)	≤278.7	279.5–363.0	363.5–445.7	446.4–713.7	≥714.5	0.06
n of cases/controls	38/67	37/68	24/82	36/69	22/83	
Multivariate adjusted model**	1 (Reference)	1.0(0.6, 1.8)	0.6 (0.3, 1.1)	1.0 (0.6, 1.9)	0.5 (0.3 0.9)	
Vitamin D (μgram/d)	≤12.2	112.3–15.9	5.9–20.4	20.4–27.3	≥27.5	0.002
n of cases/controls	46/59	33/72	29/77	24/81	25/80	
Multivariate adjusted model**	1 (Reference)	0.6 (0.3, 1.1)	0.5 (0.3, 0.9)	0.4(0.2, 0.7)	0.4 (0.2, 0.8)	
α-tocopherol (mg/d)	≤6.8	6.8–8.2	8.2–9.3	9.3–10.6	≥10.6	<0.0001
n of cases/controls	54/51	40/65	21/85	26/79	16/89	
Multivariate adjusted model**	1 (Reference)	0.6 (0.3, 1.0)	0.2 (0.1, 0.4)	0.3 (0.2, 0.6)	0.2 (0.1, 0.3)	
Vitamin C (mg/d)	≤94,5	94.6–123.2	123.3–147.9	148.1–184.6	≥184.9	0.04
n of cases/controls	45/60	30/75	26/80	31/74	25/80	
Multivariate adjusted model**	1 (Reference)	0.5 (0.3, 0.9)	0.4 (0.2, 0.8)	0.5 (0.3, 1.0)	0.4 (0.2, 0.8)	
N-3 fatty acid (g/d)	≤2.5	2.5–3.0	3.0–3.4	3.4–3.9	≥3.9	< 0.0001
n of cases/controls	44/61	45/60	22/84	33/72	13/92	
Multivariate adjusted model**	1 (Reference)	1.1 (0.6, 1.8)	0.4 (0.2, 0.7)	0.6 (0.4, 1.1)	0.2 (0.1, 0.4)	
β-carotene (μgram/d)	≤ 2296.2	2297.3–3489.5	3302.3–4463.6	4465.2–6023.9	≥ 6040.6	0.0004
n ofcases/controls	48/57	31/74	28/78	33/72	17/88	
Multivariate adjusted model**	1 (Reference)	0.5 (0.3, 0.9)	0.4 (0.2, 0.8)	0.6 (0.3, 1.0)	0.2 (0.1, 0.4)	
Cryptoxanthin (μgram/d)	≤115.9	116.2–197.7	199.4–328.0	328.2–562.5	≥564.6	0.67
n of cases/controls	43/62	20/85	25/81	30/75	39/66	
Multivariate adjusted model**	1 (Reference)	0.3 (0.2, 0.7)	0.5 (0.2, 0.8)	0.6 (0.3, 1.0)	0.9 (0.5 1.6)	

CI, confidence interval

*Energy adjustment was performed according to the residual model

**Adjusted for smoking history (past and current smokers vs non-smokers), age (years, continuous), sex (male or female), chronic disease history (presence of any of the following: hypertension, diabetes, hyperlipidemia, cardiovascular disease, stroke; yes or no), supplement use (yes or no), and alcohol consumption (yes or no).
